# Identification and Management of Chronic Pain in Primary Care: a Review

**DOI:** 10.1007/s11920-015-0659-9

**Published:** 2016-01-28

**Authors:** Sarah Mills, Nicola Torrance, Blair H. Smith

**Affiliations:** Division of Population Health Sciences, University of Dundee, Mackenzie Building, Kirsty Semple Way, Dundee, DD2 4BF Scotland UK

**Keywords:** Chronic pain, General practice, Primary care, Multidisciplinary, Pharmacological

## Abstract

Chronic pain is a common, complex, and challenging condition, where understanding the biological, social, physical and psychological contexts is vital to successful outcomes in primary care. In managing chronic pain the focus is often on promoting rehabilitation and maximizing quality of life rather than achieving cure. Recent screening tools and brief intervention techniques can be effective in helping clinicians identify, stratify and manage both patients already living with chronic pain and those who are at risk of developing chronic pain from acute pain. Frequent assessment and re-assessment are key to ensuring treatment is appropriate and safe, as well as minimizing and addressing side effects. Primary care management should be holistic and evidence-based (where possible) and incorporates both pharmacological and non-pharmacological approaches, including psychology, self-management, physiotherapy, peripheral nervous system stimulation, complementary therapies and comprehensive pain-management programmes. These may either be based wholly in primary care or supported by appropriate specialist referral.

## Introduction

Chronic pain is a common condition in primary care and one that challenges both the distinction between mind and body and the concept of cure being the goal of medical intervention.

Pain is a complex biopsychosocial phenomenon which manifests, according to the International Association for the Study of Pain (IASP) definition, as “an unpleasant sensory and emotional experience associated with actual or potential tissue damage or described by the patient in terms of such damage”[1]. IASP further defines chronic pain as “pain which has persisted beyond normal tissue healing time”. While the shift from acute to chronic pain is, rather arbitrarily, placed at 12-week duration, the main differentiation in management is that in acute pain, the focus is on addressing the cause of the pain, while in chronic pain management, the focus is on addressing the effects of the pain and maximizing function and quality of life. The current International Classification of Diseases (ICD) does not code chronic pain as a distinct diagnosis. However, proposals for ICD-11 include a code for “chronic pain” and for its subgroups, defining chronic pain as a distinct clinical entity as opposed to a result of other clinical conditions [2].

Estimates of the population prevalence of chronic pain vary widely, with between 8 and 45 % of the population reporting chronic pain, and between 10 and 15 % of the population presenting to their general practitioner (GP) [3]. The prevalence of chronic pain increases with age.

With pain affecting 100 million Americans [4], 25 million of whom report chronic daily pain [5], at an estimated economic cost of $560–635 billion/year [4, 6], chronic pain is one of the most important issues in both medicine and public health. Because of the relative newness of pain medicine as an independent subspecialty, the existence of multiple pain professional organizations and the increasing demands on the service, pain management risks are being inconsistent and uncoordinated [4]. It has been argued that “the system of pain care delivery in the United States has not kept pace with societal needs or the public’s expectations for accessible, quality pain care” [4]. Addressing chronic pain in a general practice setting has the potential to be the solution to delivering high-quality, readily accessible pain management which is available to the population in the volume required; however, inherent to that solution are the challenges posed by identifying and managing chronic pain within the constraints of general/family practice.

Dubois et al. in their report on American pain management education concluded that “pain care in America [is] fragmented, inconsistent, and incomplete, with uneven access and disparate quality” in which “undertreatment and disparities in care have been repeatedly demonstrated”[4]. In the USA, only 52 % of patients with chronic pain are managed in primary care, with the rest relying on specialist care providers [4]. Given that “the supply of pain specialists is exceeded greatly by the demand”[4] and that primary care represents a more cost-effective mechanism for healthcare delivery [7], increasing the volume of patients managed in primary care could be a crucial step to delivering the coordinated and consistent care patients with chronic pain require. The Institute of Medicine, established that “addressing the nation’s enormous burden of pain will require a cultural transformation in the way pain is understood, assessed and treated” [8].

Chronic pain has wide-reaching personal, social and psychological impacts, as well as national economic consequences. The report “Pain in Europe” demonstrated that, most people who experience chronic pain live with it for at least 7 years and that one in six chronic pain sufferers say that their pain is sometimes so bad that they want to die [9]. Of participants surveyed, 27 % said that they were less able or unable to maintain relationships with friends and family and over 40 % of chronic pain sufferers say their pain impacts on everyday activities [9]. Breivik more recently demonstrated that, in patients with chronic pain, 21 % had been diagnosed with depression because of their pain, 61 % were less able or unable to work outside the home, 19 % had lost their job and 13 % had changed jobs because of their pain [10]. It is estimated that 40–60 % of patients with chronic pain have inadequate management of their pain [9, 10].

It is of great relevance to primary care that chronic pain is also associated with significant increase in morbidity and mortality [11, 12], with 20–50 % of chronic pain sufferers having co-morbid depression [9, 13, 14•]. In an important study on multimorbidity, Barnett et al. established that chronic pain is one of the commonest morbidities to co-occur with other long-term conditions, but also that 88 % of patients with chronic pain have other chronic illnesses [9, 13, 14•]. The most common comorbidities were cardiovascular disease and depression. This high prevalence of comorbidities in patients with chronic pain can limit the applicability and utility of clinical guidelines to multimorbid patients with chronic pain [15] and restrict what treatment options can be used in pain control. Further research on managing chronic pain in multimorbid patients is essential to optimise improvements in health status, functioning, and quality of life and possibly also improve the management of their other major chronic health conditions [16]. Recent research has demonstrated a link between chronic pain and mortality. Torrance et al. established that severe chronic pain was significantly associated with all-cause mortality and particularly death from cardiovascular disease [11]. Such evidence suggests that in assessing patients with chronic pain, physicians should view chronic pain as a serious risk marker for premature mortality [11, 14•].

Primary care is “first-contact, accessible, continued, comprehensive and coordinated care”[17]. In most countries, of the 20 % of the general population who experience chronic pain, the overwhelming majority are managed in primary care by their family doctor (general practitioner, GP), while only 0.5–2 % [9] are ever referred to secondary care for pain management. Consultations on pain account for 22 % of all primary care consultations [12], and pain is one of the main reasons for patients seeking contact with healthcare [18]. Patients with chronic pain visit their GP twice as often as patients without chronic pain [19]. They also have a considerably higher level of use of emergency and unscheduled care than patients without chronic pain [20, 21] risking a care plan dictated by short-term decision making rather than comprehensive oversight of their pain as a whole [22].

GPs see undifferentiated illness in patients whom they assess, diagnose and manage in the space of a 10-minute appointment. The successful management of chronic pain in primary care relies on a multidisciplinary and holistic approach aimed at both minimizing pain as much as possible and teaching patients how to live well with chronic pain. Addressing chronic pain in primary care avoids the risk of “professionals from disparate backgrounds…[offering] treatments based on their specialty skill sets instead of providing the comprehensive multidisciplinary pain care that many patients need”[4]. For any advances in identification and management of chronic pain to be useful in a primary care setting, they must be useable within the time and resource constraints and restrictions that are inherent to general practice.

### Identification of Chronic Pain in Primary Care

In managing patients with chronic pain in primary care, the aim is generally to rule out treatable and modifiable causes and then support the patient to live as well as possible, with the maximum quality of life in spite of their chronic pain. This support takes the form of drug, non-drug and self-management interventions.

The assessment and management of chronic pain in general practice is challenging because of its complex multimodal nature (including physical, psychological and social factors) and restrictions imposed by available time and resources [23]. Initial assessment should be holistic and includes an evaluation of the severity, impact and type of pain the patient is experiencing [24•]. GPs must therefore move beyond a search for identifiable and treatable pathology, though this is also important.

#### Screening Tools and Brief Interventions

While there is no substitute for robust clinical assessment, screening tools can be a useful way to identify patients at risk of severe chronic pain or complications of chronic pain and to inform their management (Table 1). Some screening tools for specific kinds of chronic pain have been shown to be effective in primary care. The STaRT Back Tool was developed in UK primary care and is designed to help physicians in the assessment of patients at risk of progressing from acute to chronic low back pain (LBP) [25]. The tool consists of nine questions addressing known risk factors. It stratifies patients into low, medium and high risk and recommends appropriate treatment for each patient based on their risk level [25]. The STaRT Back Tool is available free-of-charge at http://www.keele.ac.uk/sbst/. Traeger et al. have also been developing and validating a screening tool which can help identify which individuals are at risk of progressing from acute pain to chronic pain among those presenting with acute LBP [26]. Tools such as the Leeds Assessment of Neuropathic Symptoms and Signs (LANSS), the Neuropathic Pain Diagnostic Questionnaire (DN4) the Neuropathic Pain Questionnaire (NPQ), ID Pain and PainDETECT are useful in determining whether pain is likely to be neuropathic, an important factor in determining subsequent management [27]. Other tools such as the Hospital Anxiety and Depression Scale can be useful in screening patients with chronic pain for other associated psychosocial comorbidities in the context of a holistic assessment [28].

**Table 1:** Tools for assessing, classifying and predicting pain [25] [27, 73–75]

Tool	Specifics	Advantages	Disadvantages
STaRT Back	Free-of-charge. Consists of nine questions addressing known risk factors for progression from acute to chronic pain.	Clinically relevant and useful. Stratifies patients into low, medium and high risk. The tool recommends appropriate treatment.	Only applicable to back pain, not generalizable
Leeds assessment of neuropathic symptoms and signs (LANSS)	Incorporates sensory description and bedside examination of sensory dysfunction. Contains 5 questions on symptoms and 2 clinical examination points.	Provides immediate information, increasing its utility in the clinical setting. Simple scoring system. Clinically validated.	The scoring simplicity may affect its discriminating ability. Not designed as a pain measurement tool. Does not take “numbness” into account as a symptom.
Neuropathic Pain Diagnostic Questionnaire/Douleur Neuropathique en 4 questions (DN4)	A clinically administered questionnaire consisting of 4 main questions (two addressing symptoms and two addressing sensory signs) with a total of 10 sub-points. Of those 10 sub-points, 7 items are based on symptoms and 3 on clinical examination.	Covers sensory descriptors and clinical signs. Covers 10 aspects of pain. Easiest tool to score, making it clinically very useful.	More detailed than LANSS but may take longer to administer. Originally produced in French, the English version has not been validated, and its scoring system is based on the original French questionnaire.
PainDETECT	Based on patient’s self-reported symptoms in a questionnaire covering 9 items.	No clinical examination required, making it easier to administer, less invasive, and possible to be delivered by non-clinicians. Makes it possible to determine the percentage of neuropathic pain in ‘total pain’.[76]	Originally developed in German; the English version has not been validated. Does not take into account clinical examination findings, potentially missing important clinical information.
Neuropathic Pain Questionnaire (NPQ)	Contains 12 items; 10 related to sensations and 2 related to patient affect	No clinical examination required.	Does not take into account clinical examination findings.
ID Pain	Covers 5 items of sensory description. Contains one clinical question clarifying if pain is related to joints.	No clinical examination required.	Does not include clinical information.
Brief Pain Inventory	A 9 question self-reported questionnaire covering pain, its management and its impact on the patient’s life	Takes into account the efficacy of current treatment and impact on the patient’s life as well as the physical symptoms. Useful measurement tool to assess pain over time during treatment.	Covers all pain rather than assessing neuropathic or nociceptive pain separately.

Current research aims to develop effective screening tools and brief intervention techniques that can be used by GPs in consultations. There is ongoing research to determine whether the addition of patient-focused pain biology education to clinical guideline-based care reduces LBP intensity at 3 months and prevents their progression to chronic LBP [29]. New research suggests that a model of brief intervention, in which clinicians give involved feedback on behaviour and how to reduce pain-generating behaviour, is successful in reducing chronic pain from medication overuse headaches [30]. These developments have the potential not only to aid GPs in identifying patients at risk of chronic pain but also in successfully targeting early interventions aimed at reducing progression to severe chronic pain.

### Management of Chronic Pain in Primary Care

Assessment and re-assessment are essential in all stages of managing chronic pain, and it is important to ensure that any new treatment is given a full and proper trial, so that potentially effective treatments are often not discarded because of an incomplete trial of treatment, inappropriate timing or dose of drugs, poor medication compliance and/or mismatched patient expectations [31]. Particularly, when treatments have been tried at the onset of chronic pain, patients may consider them not to have worked because they did not cure the pain; however, with the continuation of chronic pain and the shifting of treatment aims from cure to management, patients may find previously discounted treatments of benefit. Management should incorporate both pharmacological and non-pharmacological approaches.

It is useful for GPs and patients to discuss and agree on treatment goals before initiating treatment in order to have objective standards against which to assess treatment success or failure. An overall reduction of pain intensity by 30–50 % is formally considered to be a successful outcome, as is an overall improvement in quality of life [32]. It should be made clear to patients that achieving complete freedom from pain is an unusual outcome.

#### Relevant Guidelines

A number of recent comprehensive guidelines focus on the management of chronic pain and are relevant to primary care. These include the 2013 Scottish Intercollegiate Guidelines Network (SIGN) guideline “Management of chronic pain” (24), the 2–13 National Institute for Health and Care Excellence (NICE) guidelines “Neuropathic pain—pharmacological management” [33, 34], the 2009 American “Clinical Guidelines for the Use of Chronic Opioid Therapy in Chronic Noncancer Pain”[35], the 2007 Canadian Pain Society (CPS) guidelines for chronic neuropathic pain [36], the 2015 IASP Neuropathic Pain Special Interest Group (NeuPSIG) recommendations on “Pharmacotherapy for neuropathic pain in adults” [37•], the 2014 NICE clinical guideline “Osteoarthritis: care and management in adults” [38] and the 2013 British Pain Society (BPS) “Guidance on the Management of Pain in Older People” [39] and 2010 “Opioids for persistent pain” [40]. The SIGN Guideline is a particularly robust tool for primary care physicians; a summary of its recommendations is in Table 2 [41].

**Table 2:** Summary of some of the recommendations for chronic pain management made in the SIGN guideline.[77] Reproduced from “Managing chronic pain in the non- specialist setting: A new SIGN guideline”

Area addressed by key question	Summary of key recommendations	Level of evidence**
Assessment and planning of care	In order to best direct treatment options, a comprehensive biopsychosocial assessment, including identification of pain type (e.g. neuropathic) should be carried out in any patient with chronic pain.	GPP
Supported self-management	Self-management can be used from an early stage in a pain condition, with patients being directed to self-help resources at any stage in the patient journey.	GPP
Pharmacological therapies	There should be at least annual assessment of patients on pharmacotherapy for chronic pain.	GPP
	Tricyclic antidepressants should not be used for the management of pain in patients with chronic low back pain.	A
	Amitriptyline (25 to 125 mg/day) should be considered for the treatment of patients with fibromyalgia and neuropathic pain (excluding HIV-related neuropathic pain).	A
	Strong opioids should be considered for chronic low back pain or osteoarthritis and only continued if there is ongoing pain relief.	B
	Specialist advice or referral should be considered if there are concerns about rapid opioid dose elevation or if >180 mg/day morphine equivalent dose is needed	D
Psychologically based interventions	Consideration should be given for referral to a pain management programme for patients with chronic pain	C
	There should be an awareness of the impact of healthcare workers behaviour, as well as the treatment environment, in reinforcing unhelpful responses.	GPP
Physical therapies	Any form of exercise or exercise is recommended in for patients with chronic pain.	B
	In addition to exercise therapy, advice to stay active should be given to patients with chronic low back pain. This will improve disability in the long term. Advice alone is insufficient.	A
Complementary therapies	Acupuncture should be considered for short term relief of pain in patients with chronic low back pain or osteoarthritis.	A

^a^This is not a comprehensive list. In total, 55 graded Recommendations are included in the Guideline

^b^The grade of recommendation relates to the strength of the supporting evidence on which the evidence is based. It does not reflect the clinical importance of the recommendation. Grade A is strongest; Grade D weakest; Good practice points (GPP) represent recommended best practice based on the clinical experience of the guideline development group

Reproduced by permission of the British Journal of General Practice. Smith BH, Hardman JD, Stein A, et al. Managing chronic pain in the non-specialist setting: a new SIGN guideline. Br J Gen Pract 2014; DOI: 10.3399/bjgp14X680737

#### Drug Interventions

In their large-scale study of chronic pain, Breivik found that almost half of all people with chronic pain were taking non-prescription analgesics, including non-steroidal anti-inflammatory drugs (NSAIDs) (55 %), paracetamol (acetaminophen) (43 %) and weak opioids (13 %). Two thirds were taking prescription medicines, including NSAIDs (44 %), weak opioids (23 %), paracetamol (acetaminophen) (18 %), COX-2 inhibitors (1–36 %) and strong opioids (5 %) [10].

Given its good tolerability and efficacy, paracetamol has long represented a good baseline drug for management of chronic pain [42]. However, recent studies call this into question, and we need a degree of circumspection about even this basic treatment [16, 43]. Additional analgesics should be targeted to the type of pain. While pain type is broadly either nociceptive or neuropathic, many pains are mixed, requiring multi-modal pharmacology [43].

Pain that is predominantly neuropathic should be treated with baseline regular paracetamol, followed by progression through tricyclic antidepressants, then gabapentin and then pregabalin [34]. Topical preparations, including lidocaine and capsaicin, can be effective in managing neuropathic pain and should be considered in patients where pain is very localized and/or first-line treatments are contraindicated or have been insufficient. While tramadol can be initiated in primary care, it is recommended that morphine only be initiated for neuropathic pain by secondary care physicians [44]. Patients requiring strong opiates for neuropathic pain should be considered for referral to secondary care pain management services.

For pain that is predominantly nociceptive, paracetamol should be trialed in the first instance and then augmented as appropriate with NSAIDs, such as ibuprofen and naproxen, where no contra-indications exist. NSAIDs should only be used after taking into account age and comorbidities (including asthma, chronic kidney disease and risk of GI bleeding). Current research suggests that inappropriate NSAID prescribing is common and needs to be addressed urgently to improve patient safety [45]. Topical NSAIDs, such as ibuprofen gel, can be used effectively in many causes of chronic pain and have better safety than oral NSAIDs and comparable efficacy; however, current evidence does not support their use in back pain [46].

Use of opioids can be considered in patients with chronic pain; however, extreme caution is needed. It is important to note that prescribing above 120-mg morphine equivalent dose per day requires specialist supervision [35]. When evaluating potential use of opioids, it is important to bear in mind that there is no evidence for their effectiveness in long-term use and that there are risks of potentially serious adverse effects including drowsiness, constipation, endocrine, respiratory depression and even death. Compounding both these factors is the risk of clinical dependence and addiction, meaning that once patients have been started on opioids, it can be challenging to discontinue their use [35].

Patients should have their analgesia reviewed at each assessment, in order to titrate medications to their maximum effective or tolerated dose, assess the level of analgesia produced, and withdraw any medications that have not produced the desired therapeutic effect. As patients’ analgesia requirements will change over time, regular on-going medication reviews are required to ensure that the medication continues to be appropriate, that the analgesia is achieving the best clinical outcome possible and that patients are not experiencing side effects.

### Non-Drug or Complex Interventions

Breivik’s evaluation of patients with chronic pain demonstrated that one third of chronic pain sufferers were not currently using any treatment and that two thirds were using non-medication treatments, including massage (30 %), physical therapy (21 %) and acupuncture (13 %) [10].

#### Psychological Approaches

Cognitive-behavioural therapy (CBT) has been used in primary care both on its own and as part of a comprehensive pain management programme (PMP). A recent randomized control trial (RCT) has demonstrated that training healthcare professionals on a 2-day course allowed them to deliver a CBT-based intervention in primary care settings to patients with low back pain [47]. Participants who received CBT had improved measures of disability, reduced pain intensity, reduced depression and better quality of life 1 year after intervention than those receiving standard care [48]. More recent evidence suggests that acceptance and commitment therapy (ACT) can be as effective as CBT in patients with chronic pain [49]. A recent Cochrane review of 35 trials examining psychological approaches to chronic pain management found that behaviour therapy produced small short-lived benefits, but that patients who had CBT showed improvements in pain, as well as in disability, mood and catastrophic thinking [50].

#### Self-Management

Self-management has been defined as activities which “enhance function, improve mood and decrease pain” by targeting and challenging the “emotional, cognitive and behavioural responses to pain” [51]. There is evidence that supported self-management, for example self-management programmes and electronic delivery including online resources, is effective to complement other therapies [24•]. For example, MoodJuice has been developed as a freely available Chronic Pain Toolkit and Self Help Guide (http://www.moodjuice.scot.nhs.uk/)) which can be accessed by patients and which can form a useful framework for self-management in chronic pain. The Pain Toolkit (http://www.paintoolkit.org) is another well-established resource. Boyers et al. determined that self-management is effective and potentially cost-effective in improving pain among an older adult population, though noted that further research was required [52].

#### Physiotherapy

For musculoskeletal pain, current research confirms the beneficial effect of physiotherapy on both pain intensity and on physical function [53]. A recent systematic review and meta-analysis in physiotherapy for chronic pain demonstrated evidence for the effectiveness of adding motivational interventions to traditional physiotherapy in terms of increasing physical activity and patient adherence to physiotherapy exercises [54].

#### Peripheral Nervous System Stimulation

Trans-cutaneous electronic nerve simulation (TENS) is a pain relief treatment which is based on the gate-control theory of pain and which delivers recurrent stimulation to neurons via electrodes placed on the skin. TENS has been shown to have a positive analgesia outcome (24) and is a patient-centered approach in that patients are able to control the frequency, intensity and duration of treatment. One recent study showed that TENS proved beneficial in 70 % of patients over the course of 2 months and that TENS-responsive patients decreased pain intensity scores by a mean of 9.8 on a 0–100 mm scale [55]. External noninvasive peripheral nerve stimulation (EN-PNS) is a novel form of peripheral nerve stimulation that is currently under development and uses an external nerve-mapping probe that is placed on the skin and connected to a power source [56]. Initial clinical trial results indicate the potential for huge benefit, though the sample size was small [56].

#### Complementary Therapies

Complementary and alternative medicine (CAM) is a commonly used addition to pain control regimens. One recent survey demonstrated that over 70 % of patients with chronic pain had used complementary therapies and that levels of patient satisfaction were higher in the group using CAM [57]. The complementary therapies most commonly used for chronic pain are osteopathy, chiropractic, homeopathy, acupuncture and herbalism (and rarely hypnosis and aromatherapy) [58, 59]. Most patients using alternative medicine do so in conjunction with conventional healthcare [58]. There is good evidence in favour of the use of acupuncture leading to its use being recommended for patients with osteoarthritis or chronic low back pain, but limited or absent evidence for the effectiveness of other CAM treatments [24•]. The 2010 NICE guidelines for LBP advocate the use of spinal manipulation [60]. However, a more recent systematic review of 26 RCTs found that, while there is high-quality evidence demonstrating that spinal manipulation has a small and significant benefit on pain levels and functional status, there are insufficient data to determine whether this benefit is clinically relevant or to gauge its effects on outcomes such as return-to-work, quality-of-life and financial implications of care [61].

#### Comprehensive Pain Management Programs

PMPs are effective in both primary and secondary care [23]. One study demonstrated that individuals engaged in PMPs in primary care required less medication and had lower healthcare utilization over the following 3 years [62]. In their new guidelines, the BPS advocates the use of PMPs, based on cognitive behavioral principles, as the treatment of choice for people with persistent pain with biopsychosocial dysfunction [63]. However, in practice, access to PMPs is geographically restricted, limiting their utility in primary care pain management.

### Emerging Research in Chronic Pain

#### Pharmacist Roles in Prescribing

Pharmacists have expertise in therapeutics and polypharmacy, and new research demonstrates that pharmacist prescribing in primary care has the potential to confer additional benefit over medication review alone [64]. It has been suggested that expanding pharmacists’ roles to include the provision of information, discussing barriers to pain treatment, monitoring pain disability and appropriately managing pharmacotherapy optimizes the effectiveness of pharmacological interventions for chronic pain and minimize adverse effects experienced by patients [65].

#### Collaborative Intervention

Collaborative interventions, where the therapeutic relationship between patients and doctors is emphasized, have been shown to produce modest but statistically significant improvement in outcomes in patients with chronic pain [66]. Research exploring interventions targeted at both clinicians and patients demonstrated improved management in patients with chronic pain [67].

#### Mindfulness in Chronic Pain

Mindfulness, an approach aimed at developing beneficial reactions to both mental and physical processes that contribute to dysfunctional behaviour and emotional distress, has featured as a tool in emerging research in chronic pain management. McCubbin et al. demonstrated that mindfulness was associated with improvements in patient-centered outcomes for over a year after initial intervention and with a reduced utilization of healthcare services for up to 18 months [68]. In their study of Mindfulness Based Functional Therapy (MBFT) in primary care, Schutze et al. showed good patient adherence and satisfaction, reduced pain catastrophising and improved physical functioning among people with chronic LBP [69]. In spite of a number of recent studies in this area, however, in their meta-analysis, Bawa et al. demonstrated only limited evidence in support of mindfulness-based interventions for patients with chronic pain and called for more and better studies in this area [70, 71].

#### Telecare

The use of telecare in patients with chronic pain is growing an evidence base suggesting it is beneficial in chronic pain management. Kroenke et al. demonstrated that telecare doubled the number of patients who achieved a reduction in their pain of over 30 %. This pain reduction was sustained at 12 months [72].

#### Decision Support Tool for Clinicians

The BPS has produced patient care pathways for chronic pain, in collaboration with “Map of Medicine”, intended for use in primary care. These decision maps are tools which are available online to any healthcare professional, as well as members of the public, via http://bps.mapofmedicine.com/evidence/bps/index.html. They are designed to present evidence-based medicine in clinical flowcharts to help clinicians apply the best evidence to the patient in front of them. They have developed five evidence-based pain pathways covering chronic pelvic pain, chronic widespread pain including fibromyalgia, neuropathic pain, low back and radicular pain and initial assessment and early management of pain. Such interactive flowcharts have the potential to improve the management of chronic pain.

## Conclusion

Given the frequency of chronic pain as a presentation in primary care, and that the vast majority of patients with chronic pain are managed in primary care, GPs should have adequate evidence, training and resources to assess and manage chronic pain. Recent high-quality guidelines are available, and some trials show encouraging results for the application of pharmacological and non-pharmacological interventions in the holistic management of patients with chronic pain. However, there continues to be a relative lack of high-quality primary-care-focused research in chronic pain. Further education, research and resourcing targeted at primary care management of chronic pain is required to ensure that the care being delivered is as efficient, effective and evidence-based as possible.

## References

[1] Pain IAftSo. IASP Taxonomy - IASP. 2015 [cited 2015 August 6]. Available from: http://www.iasp-pain.org/Education/Content.aspx?ItemNumber=1698&navItemNumber=576.

[2] Treede RD, Rief W, Barke A, Aziz Q, Bennett MI, Benoliel R (2015). A classification of chronic pain for ICD-11. Pain.

[3] McQuay HJKE, Moore RA (2008). Epidemiology of chronic pain.

[4] Dubois MY, Follett KA (2014). Pain medicine: the case for an independent medical specialty and training programs. Acad Med J Assoc Am Med Coll.

[5] Nahin RL (2015). Estimates of pain prevalence and severity in adults: United States, 2012. J Pain.

[6] Gaskin DJ, Richard P (2012). The economic costs of pain in the United States. J Pain.

[7] Davis K SK, Schoen C, Squires D. <Mirror, Mirror on the Wall. 2014 Update: How the U.S. Health Care System Compares Internationally - The Commonwealth Fund.pdf>. The Commonwealth Fund.

[8] Medicine Io. Relieving Pain in America: A Blueprint for Transforming Prevention, Care, Education, and Research. [book]. Washington, DC: The National Academies Press; 2011. 22553896

[9] Breivik H, Collett B, Ventafridda V, Cohen R, Gallacher D (2006). Survey of chronic pain in Europe: prevalence, impact on daily life, and treatment. Eur J Pain.

[10] Breivik H (2012). A major challenge for a generous welfare system: a heavy socio-economic burden of chronic pain conditions in Sweden—and how to meet this challenge. Eur J Pain (Lond. Engl).

[11] Torrance N, Elliott AM, Lee AJ, Smith BH (2010). Severe chronic pain is associated with increased 10 year mortality. A cohort record linkage study. Eur J Pain.

[12] Mäntyselkä PT, Turunen JHO, Ahonen RS, Kumpusalo EA (2003). Chronic pain and poor self-rated health. JAMA.

[13] Donaldson SL. 150 years of the Annual Report of the Chief Medical Officer: On the state of public health 2008. Department of Health, Richmond House, 79 Whitehall, London SW1A 2NJ, UK, dhmail@dh.gsi.gov.uk; 2009.

[14.•] Barnett K, Mercer SW, Norbury M, Watt G, Wyke S, Guthrie B (2012). Epidemiology of multimorbidity and implications for health care, research, and medical education: a cross-sectional study. Lancet.

[15] Guthrie B, Payne K, Alderson P, McMurdo MET, Mercer SW (2012). Adapting clinical guidelines to take account of multimorbidity. Br Med J.

[16] Butchart A, Kerr EA, Heisler M, Piette JD, Krein SL (2009). Experience and management of chronic pain among patients with other complex chronic conditions. Clin J Pain.

[17] Organisation WH. Primary Health Care, Main terminology: World Health Organization; 2015 [cited 2015 August 6]. Available from: http://www.euro.who.int/en/health-topics/Health-systems/primary-health-care/main-terminology.

[18] Briggs EV, Carr ECJ, Whittaker MS (2011). Survey of undergraduate pain curricula for healthcare professionals in the United Kingdom. Eur J Pain.

[19] Andersson HIEG, Leden I, Schersten B (1999). Impact of chronic pain on health care seeking, self care, and medication. Results from a population-based Swedish study. J Epidemiol Community Health.

[20] Eriksen JSP, Ekholm O, Rasmussen NK (2004). Health care utilisation among individuals reporting long-term pain: an epidemiological study based on Danish National Health Surveys. Eur J Pain.

[21] Cordell WHKK, Giles BK, Jones JB, Jones JH, Brizendine EJ (2002). The high prevalence of pain in emergency medical care. Am J Emerg Med.

[22] McLeod D, Nelson K. The role of the emergency department in the acute management of chronic or recurrent pain. Australas Emerg Nurs J. 2012. 10.1016/j.aenj.2012.12.001 23622554

[23] Smith BH, Torrance N (2011). Management of chronic pain in primary care. Curr Opin Support Palliat Care.

[24.•] Network SIG. SIGN 136 Management of Chronic Pain. In: Scotland HI, editor.: Health Improvement Scotland; December 2013. **An important work allowing a streamlined evidence-based approach to managing chronic pain as a distinct primary care condition. Internationally relevant. Readily accessible and easy to integrate into clinical consultations.**

[25] Beneciuk JM, Bishop MD, Fritz JM, Robinson ME, Asal NR, Nisenzon AN (2013). The STarT back screening tool and individual psychological measures: evaluation of prognostic capabilities for low back pain clinical outcomes in outpatient physical therapy settings. Phys Ther.

[26] Traeger A, Henschke N, Hubscher M, Williams CM, Kamper SJ, Maher CG (2015). Development and validation of a screening tool to predict the risk of chronic low back pain in patients presenting with acute low back pain: a study protocol. BMJ Open.

[27] Haanpaa ML, Backonja MM, Bennett MI, Bouhassira D, Cruccu G, Hansson PT (2009). Assessment of neuropathic pain in primary care. Am J Med.

[28] Fitzpatrick R, Gibbons E, Mackintosh A. An overview of patient-reported outcome measures for people with anxiety and depression. Oxford: University of Oxford, 2010.

[29] Traeger AC, Moseley GL, Hubscher M, Lee H, Skinner IW, Nicholas MK (2014). Pain education to prevent chronic low back pain: a study protocol for a randomised controlled trial. BMJ Open.

[30] Kristoffersen ES, Straand J, Vetvik KG, Benth JS, Russell MB, Lundqvist C (2015). Brief intervention for medication-overuse headache in primary care. The BIMOH study: a double-blind pragmatic cluster randomised parallel controlled trial. J Neurol Neurosurg Psychiatry.

[31] Smith BH, Hopton JL, Chambers WA (1999). Chronic pain in primary care. Fam Pract.

[32] Dworkin RH, Turk DC, Wyrwich KW, Beaton D, Cleeland CS, Farrar JT (2008). Interpreting the clinical importance of treatment outcomes in chronic pain clinical trials: IMMPACT recommendations. J Pain.

[33] NICE NIfHaCE. Neuropathic pain—pharmacological management. NICE clinical guideline 173. 2013(November 2013):1–43.

[34] NICE NIfH, Excellence C. Neuropathic pain:the pharmacological management of neuropathic pain in adults in nonspecialist settings 2010. CG98:[Available from: https://www.nice.org.uk/guidance/cg96/resources/guidance-neuropathic-pain-the-pharmacological-management-of-neuropathic-pain-in-adults-in-nonspecialist-settings-pdf.

[35] Chou R FG, Fine PG, et al. Clinical Guidelines for the Use of Chronic Opioid Therapy in Chronic Noncancer Pain. J Pain 2009; 10;10:113–30 10.1016/j.jpain.2008.10.008 PMC4043401 19187889

[36] Moulin DE, Clark AJ, Gilron I, Ware MA, Watson CP, Sessle BJ (2007). Pharmacological management of chronic neuropathic pain—consensus statement and guidelines from the Canadian Pain Society. Pain Res Manag J Can Pain Soc J de la societe canadienne pour le traitement de la douleur.

[37.•] Finnerup NB, Attal N, Haroutounian S, McNicol E, Baron R, Dworkin RH (2015). Pharmacotherapy for neuropathic pain in adults: a systematic review and meta-analysis. Lancet Neurol.

[38] Nice NIfH, Care E. Osteoarthritis. In: Guidelines C, editor. 2014.

[39] Abdulla A, Adams N, Bone M, Elliott AM, Gaffin J, Jones D (2013). Guidance on the management of pain in older people. Age Ageing.

[40] Society TBP. Opioids for persistent pain. 2010.

[41] Smith BHHJ, Stein A, Colvin L (2014). Managing chronic pain in the non-specialist setting: a new SIGN guideline. Br J Gen Pract.

[42] Organisation WH. WHO | WHO's cancer pain ladder for adults. WHO. 2013.

[43] Bennett MI, Smith BH, Torrance N, Lee AJ (2006). Can pain can be more or less neuropathic? Comparison of symptom assessment tools with ratings of certainty by clinicians. Pain.

[44] Excellence NIfHaC. Neuropathic pain—pharmacological management. NICE Clinical Guideline 173. 2013;173.

[45] Ussai S, Miceli L, Pisa FE, Bednarova R, Giordano A, Della Rocca G (2015). Impact of potential inappropriate NSAIDs use in chronic pain. Drug des Dev Ther.

[46] Haroutiunian S, Drennan DA, Lipman AG (2010). Topical NSAID therapy for musculoskeletal pain. Pain Med.

[47] Lamb SE, Lall R, Hansen Z, Withers EJ, Nichols V, Griffiths F, Potter R, Underwood M, Castelnuovo E, Szczepura A. A multicentred randomised controlled trial of a primary care-based cognitive behavioural programme for low back pain. The Back Skills Train (BeST) Trial Health Technol Assess. 2010;14 (41):1–281. 10.3310/hta14410 20807469

[48] Lamb SE, Hansen Z, Lall R, Castelnuovo E, Withers EJ, Nichols V (2010). Group cognitive behavioural treatment for low-back pain in primary care: a randomised controlled trial and cost-effectiveness analysis. Lancet.

[49] Wetherell JL, Afari N, Ayers CR, Stoddard JA, Ruberg J, Sorrell JT (2011). Acceptance and commitment therapy for generalized anxiety disorder in older adults: a preliminary report. Behav Ther.

[50] Williams AC, Eccleston C, Morley S (2012). Psychological therapies for the management of chronic pain (excluding headache) in adults. Cochrane Database Syst Rev.

[51] Cameron PSC (2012). The need to define chronic pain self-management. J Pain Manag.

[52] Boyers D, McNamee P, Clarke A, Jones D, Martin D, Schofield P (2013). Cost-effectiveness of self-management methods for the treatment of chronic pain in an aging adult population a systematic review of the literature. Clin J Pain.

[53] Magalhaes MO, Muzi LH, Comachio J, Burke TN, Franca FJR, Ramos LAV (2015). The short-term effects of graded activity versus physiotherapy in patients with chronic low back pain: a randomized controlled trial. Man Ther.

[54] McGrane N, Galvin R, Cusack T, Stokes E (2015). Addition of motivational interventions to exercise and traditional physiotherapy: a review and meta-analysis. Physiotherapy.

[55] Loh J, Gulati A (2015). The use of transcutaneous electrical nerve stimulation (TENS) in a major cancer center for the treatment of severe cancer-related pain and associated disability. Pain Med.

[56] Johnson S, Ayling H, Sharma M, Goebel A (2015). External noninvasive peripheral nerve stimulation treatment of neuropathic pain: a prospective audit. Neuromodulation.

[57] Pannek J, Pannek-Rademacher S, Wollner J (2015). Use of complementary and alternative medicine in persons with spinal cord injury in Switzerland: a survey study. Spinal Cord.

[58] Haetzman M, Elliott AM, Smith BH, Hannaford P, Chambers WA (2003). Chronic pain and the use of conventional and alternative therapy. Fam Pract.

[59] Cosio D, Lin EH (2015). Effects of a pain education program in complementary and alternative medicine treatment utilization at a VA medical center. Complement Ther Med.

[60] Underwood M, Watson P, et al. Low back pain | Guidance and guidelines | NICE. 2009;CG88.

[61] Rubinstein SM, van Middelkoop M, Assendelft WJJ, de Boer MR, van Tulder MW (2011). Spinal manipulative therapy for chronic low-back pain: an update of a Cochrane review. Spine.

[62] Westman A, Linton SJ, Öhrvik J, Wahlén P, Leppert J (2008). Do psychosocial factors predict disability and health at a 3-year follow-up for patients with non-acute musculoskeletal pain? : a validation of the Örebro Musculoskeletal Pain Screening Questionnaire. Eur J Pain.

[63] Society TBP (2013). Guidelines for pain management programmes for adults.

[64] Bruhn HBB, Christine; Elliott, Alison; Hannaford, Philip; Lee, Amanda; McNamee, Paul; Smith, Blair H; Watson, Margaret; Wright, David. Pharmacist-led management of chronic pain in primary care: results from a randomised controlled exploratory trial. 2013. 10.1136/bmjopen-2012-002361 PMC3641445 23562814

[65] Jouini G, Choiniere M, Martin E, Perreault S, Berbiche D, Lussier D (2014). Pharmacotherapeutic management of chronic noncancer pain in primary care: lessons for pharmacists. J Pain Res.

[66] Dobscha SK, Corson K, Perrin NA, Hanson GC, Leibowitz RQ, Doak MN (2009). Collaborative care for chronic pain in primary care: a cluster randomized trial. JAMA.

[67] Lalonde L, Choiniere M, Martin E, Levesque L, Hudon E, Belanger D (2015). Priority interventions to improve the management of chronic non-cancer pain in primary care: a participatory research of the ACCORD program. J Pain Res.

[68] McCubbin T, Dimidjian S, Kempe K, Glassey MS, Ross C, Beck A (2014). Mindfulness-based stress reduction in an integrated care delivery system: one-year impacts on patient-centered outcomes and health care utilization. Permanente J.

[69] Schutze R, Slater H, O'Sullivan P, Thornton J, Finlay-Jones A, Rees CS (2014). Mindfulness-based functional therapy: a preliminary open trial of an integrated model of care for people with persistent low back pain. Front Psychol.

[70] Bawa FL, Mercer SW, Atherton RJ, Clague F, Keen A, Scott NW (2015). Does mindfulness improve outcomes in patients with chronic pain? Systematic review and meta-analysis. Br J Gen Pract J R Coll Gen Pract.

[71] Toye F, Andrews J, Barker K, Seers K, Allcock N, Briggs M (2013). Patients' experiences of chronic non-malignant musculoskeletal pain: a qualitative systematic review. Br J Gen Pract.

[72] Kroenke K, Krebs EE, Wu J, Yu Z, Chumbler NR, Bair MJ (2014). Telecare collaborative management of chronic pain in primary care: a randomized clinical trial. JAMA.

[73] Bennett MI, Attal N, Backonja MM, Baron R, Bouhassira D, Freynhagen R (2007). Using screening tools to identify neuropathic pain. Pain.

[74] Mathieson S, Maher CG, Terwee CB (2015). Folly de Campos T, Lin CW. Neuropathic pain screening questionnaires have limited measurement properties. A systematic review. J Clin Epidemiol.

[75] Cleeland C. The Brief Pain Inventory User Guide. 2009.

[76] Rados I, Sakic Zdravcevic K, Hrgovic Z (2013). painDETECT questionnaire and lumbar epidural steroid injection for chronic radiculopathy. Eur Neurol.

[77] Smith BH, Hardman JD, Stein A, Colvin L (2014). Managing chronic pain in the non- specialist setting: a new SIGN guideline. Br J Gen Pract.

